# Chronic Ectopic Pregnancy Presenting as a Complex Adnexal Mass With Negative Beta-Human Chorionic Gonadotropin Mimicking Ovarian Malignancy: A Case Report

**DOI:** 10.7759/cureus.111583

**Published:** 2026-06-26

**Authors:** Enas Alwan, Anessa Thabet, Saleh Al-wageeh

**Affiliations:** 1 Department of Obstetrics and Gynecology, Ibb University, Ibb, YEM; 2 Department of Gynecology, Ibb University of Medical Science, Ibb, YEM; 3 Department of General Surgery, School of Medicine, Ibb University of Medical Science, Ibb, YEM

**Keywords:** case report, chronic, diagnosis, ectopic pregnancy, magnetic resonance imaging, pelvic mass, pregnancy

## Abstract

Chronic ectopic pregnancy (CEP) is an uncommon form of ectopic gestation characterized by a slow clinical course and nonspecific symptoms, frequently resulting in delayed or challenging diagnosis. We report the case of a 29‑year‑old woman who presented with persistent lower abdominal pain and intermittent vaginal bleeding for one month, beginning two months after a suspected spontaneous miscarriage. Physical examination revealed a left adnexal mass. Laboratory investigations demonstrated negative serum beta‑human chorionic gonadotropin (β‑hCG) and a normal cancer antigen 125 (CA‑125) level. Pelvic magnetic resonance imaging (MRI) identified a 9.8 cm complex hemorrhagic adnexal mass without post‑contrast enhancement or solid components suggestive of malignancy. Because of progressive hemoglobin decline and persistent diagnostic uncertainty, the patient underwent exploratory laparotomy. Intraoperatively, an organized hematocele with dense adhesions involving the left adnexa was identified, and a left salpingo‑oophorectomy was performed. Histopathology demonstrated chorionic villi and trophoblastic remnants within organized hemorrhagic tissue, confirming CEP. The postoperative course was uneventful, and the patient remained asymptomatic at six‑month follow‑up. This case highlights that CEP may mimic ovarian malignancy despite negative serum β‑hCG and nonspecific imaging findings. Clinicians should maintain CEP within the differential diagnosis when evaluating complex hemorrhagic adnexal masses in reproductive-aged women, even when serum β-hCG is negative, to facilitate timely diagnosis and appropriate surgical management.

## Introduction

Ectopic pregnancy is defined as implantation of a fertilized ovum outside the endometrial cavity, most commonly within the fallopian tube [1]. Patients typically present with amenorrhea, lower abdominal pain, and vaginal bleeding [2]. Chronic ectopic pregnancy (CEP) is a rare form of ectopic pregnancy, accounting for approximately 1%-2% of cases, and frequently presents a diagnostic challenge because its manifestations are often nonspecific and overlap with those of pelvic inflammatory disease, tubo‑ovarian abscess, endometriosis, or ovarian malignancy [2-5]. Delayed or missed diagnosis may result in significant morbidity, including hemorrhage, extensive pelvic adhesions, fertility impairment, or unnecessary surgical intervention [3,4].

Unlike acute ectopic pregnancy, CEP usually follows an indolent clinical course characterized by gradual trophoblastic invasion, repeated minor tubal ruptures, and progressive organization of hemorrhagic material into a pelvic hematocele [5,6]. These pathologic changes commonly contribute to delayed recognition and diagnostic uncertainty [2]. Serum beta‑human chorionic gonadotropin (β‑hCG) levels may be low or even negative because chorionic villi are often sparse, degenerated, or nonviable, a well‑recognized phenomenon in CEP that may falsely reassure clinicians and delay diagnosis [7,8]. Consequently, a negative β-hCG result does not exclude CEP, and clinicians should maintain CEP within the differential diagnosis in reproductive-aged women presenting with complex pelvic masses or persistent pelvic pain [8,9].

Imaging plays an important role in the diagnostic evaluation of CEP. Although ultrasonography remains the first‑line modality, findings are frequently inconclusive because CEP may appear as a heterogeneous adnexal or pelvic mass with extensive hemorrhagic and inflammatory changes [9]. In such cases, magnetic resonance imaging (MRI) may provide additional anatomic characterization and improve diagnostic confidence when conventional imaging and laboratory findings are equivocal [10].

Definitive diagnosis is usually established histopathologically, demonstrating hemorrhage, blood clots, tubal wall disintegration, chronic inflammatory changes, and degenerated chorionic villi [5,11]. We report a case of CEP presenting as a large complex pelvic mass mimicking ovarian malignancy in a 29‑year‑old woman two months after a suspected miscarriage. This case highlights the diagnostic value of MRI and reinforces the importance of considering CEP in the differential diagnosis despite negative β‑hCG findings.

## Case presentation

A 29‑year‑old woman, gravida 3, para 2, abortion 1, presented to the gynecology outpatient clinic in June 2026, with a one‑month history of persistent lower abdominal pain and abnormal vaginal bleeding. Two months earlier, she had experienced a suspected spontaneous miscarriage at approximately eight weeks' gestation, which was managed expectantly at home without medical follow‑up. No pregnancy testing was performed during the interval between the suspected miscarriage and presentation because the patient did not seek medical attention at that time. Following the miscarriage, she developed persistent amenorrhea. One month before presentation, she noticed gradually worsening dull lower abdominal pain associated with intermittent vaginal spotting. Unfortunately, the pain scale was not documented during the initial clinical assessment. She denied fever, nausea, vomiting, or bowel and bladder symptoms. Her past medical and surgical history was unremarkable. Risk factors for ectopic pregnancy were systematically assessed and were absent, including previous pelvic inflammatory disease, prior ectopic pregnancy, tubal surgery, intrauterine device use, smoking, and assisted reproductive techniques.

On examination, she was hemodynamically stable, with a pulse of 90 beats per minute and blood pressure of 100/60 mmHg. Abdominal examination revealed mild tenderness localized to the left iliac fossa without guarding or rebound. Pelvic examination demonstrated a full pouch of Douglas, mild cervical motion tenderness, and a firm, tender, fixed left adnexal mass estimated at 8-10 cm in diameter. The uterus was normal in size.

Laboratory investigations, summarized in Table 1, revealed a hemoglobin drop over 48 hours, indicating ongoing internal bleeding. Mild leukocytosis with neutrophilia was also observed. Serum beta-human chorionic gonadotropin (β-hCG) was negative, excluding active gestation, and cancer antigen 125 (CA-125) was within normal limits.

**Table 1:** Diagnostic laboratory data, tumor markers, and preoperative serological evaluation β-hCG: beta-human chorionic gonadotropin, BT: bleeding time, CA-125: cancer antigen 125, CT: clotting time, g/dL: grams per deciliter, Hb: hemoglobin, HBsAg: hepatitis B surface antigen, HCV: hepatitis C virus, HIV: human immunodeficiency virus, mIU/mL: milli-international units per milliliter, PCV: packed cell volume, Rh: Rhesus factor, U/mL: units per milliliter, WBC: white blood cell

Test category	Parameter (units)	Initial presentation	Preoperative	Reference range
Hematology	Hb (g/dL)	12.8	10.9	12.50-16.00
	PCV/hematocrit (%)	38.9	33.7	37.00-47.00
	WBC count (cell/mm³)	6.04	10.26	4.00-11.00
	Neutrophils (%)	54.8	78.5	40.00-70.00
	Platelet count (×10³/mm³)	283	239	150.00-450.00
Coagulation	BT (minutes)	2'30''	-	2-7
	CT (minutes)	5'45''	-	5-11
Biochemistry	Serum creatinine (mg/dL)	0.76	-	0.6-1.1
Tumor/pregnancy	Serum β-hCG (mIU/mL)	<5	-	<5 (Negative)
	CA-125 (Roche) (U/mL)	12.99	-	0.00-35.00
Serology	HBsAg	Non-reactive	-	Non-reactive
	HIV 1 and 2	Non-reactive	-	Non-reactive
	HCV	Non-reactive	-	Non-reactive
Blood group	Group and Rh type	O positive	-	N/A

Pelvic magnetic resonance imaging showed a large, well-defined midline lesion measuring 9.8 × 10.7 × 9.8 cm. The lesion exhibited heterogeneous signal with predominant T1 and T2 hyperintensity and patchy hypointense areas, suggesting septations, a hemorrhagic adnexal lesion, or internal clots. No post-contrast enhancement was seen after intravenous gadolinium administration. The mass exerted a minor mass effect on the posterior uterine wall without invasion. Both ovaries were in contact with the lesion; the right ovary contained a separate 3.7 cm simple cyst, while the left ovary appeared normal (Figure 1).

**Figure 1:**
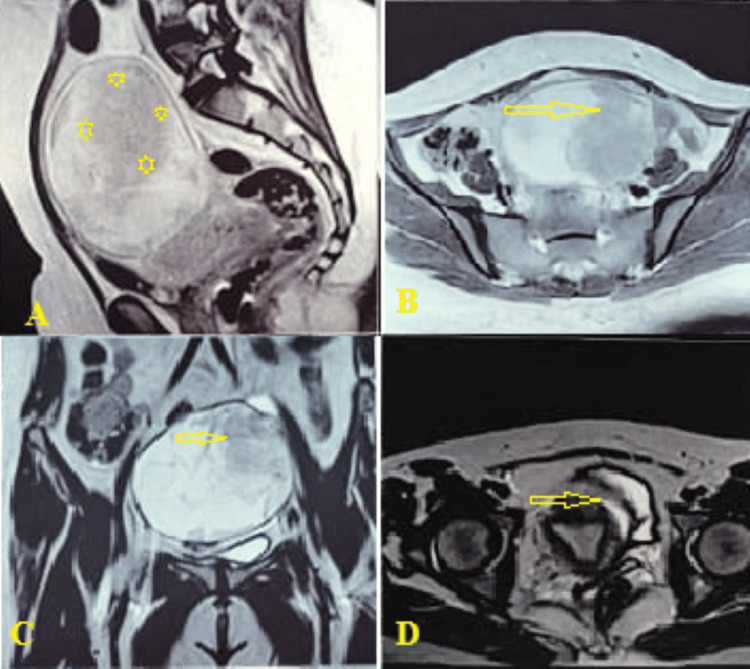
Pelvic magnetic resonance imaging (A) Sagittal T2-weighted image shows a large midline lesion measuring 9.8 × 10.7 × 9.8 cm with mass effect on the posterior uterine wall (arrow). (B) Axial T1-weighted image demonstrates a predominantly hyperintense signal within the lesion, consistent with hemorrhagic content. (C) Axial T2-weighted fat-saturated image reveals a heterogeneous signal with patchy hypointense areas (arrowheads), suggesting internal clots or septations. (D) Post-contrast T1-weighted image shows no significant enhancement within the lesion, arguing against a solid neoplasm.

After a multidisciplinary discussion, it was decided to proceed with an exploratory laparotomy due to the large mass size, progressive hemoglobin decline, diagnostic uncertainty, and the need to exclude malignancy.

Intraoperatively, approximately 500 mL of old hemoperitoneum, comprising both fresh blood and organized dark clots, was evacuated from the pelvic cavity and pouch of Douglas. A large, congested, hemorrhagic mass was identified in the left adnexal region, densely adherent to the left ovary, left fallopian tube, and broad ligament. The left fallopian tube was markedly distended and cyanotic, appearing dilated and incorporated into the mass, with evidence of chronic inflammation and fibrosis. A clear site of tubal rupture with active hemorrhage and protruding products of conception was noted. The uterus appeared grossly normal, and the contralateral adnexa were inspected and found to be unremarkable, except for a small simple cyst on the right ovary. A left salpingo-oophorectomy was performed to ensure complete excision of the mass and achieve definitive hemostasis (Figure 2).

**Figure 2:**
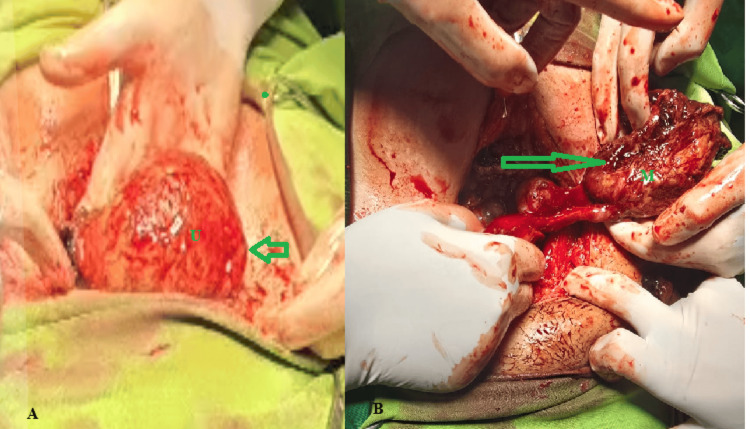
Intraoperative findings (A) A large, congested, hemorrhagic mass (M) occupies the left adnexal region, displacing the uterus (U) to the right, with a dark violaceous appearance consistent with chronic hematocele. (B) After dissection, the mass (M) appears adherent to pelvic structures, including the left ovary and fallopian tube; the right ovary (RO) is normal.

The right ovarian cyst was fenestrated, and the right ovary was preserved. The patient tolerated the procedure well, with an estimated blood loss of 500 mL.

Histopathological examination revealed a fallopian tube containing immature chorionic villi, intermediate trophoblast, blood, and fibrin, confirming CEP. Additional findings included hemorrhage, blood clots, tubal wall disintegration, multiple minor ruptures, and areas of dense fibrosis and necrosis within degenerated chorionic villi. No malignant cells were identified (Figure 3).

**Figure 3:**
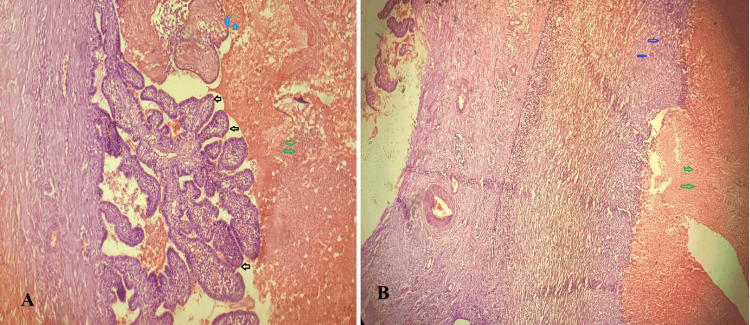
Histopathological examination of the surgical specimen (A) Low-power view (40×) showing the fallopian tube wall (black arrows) with an associated large, organized blood clot and fibrin (green arrows) and "ghosts" of chorionic villi (blue arrows). (B) High-power view (400×) revealing pathognomonic features of chronic ectopic pregnancy, including organized blood clot and fibrin (green arrows) and "ghosts" of chorionic villi (blue arrows). The digital arrows indicate degenerated "ghosts" of chorionic villi embedded within necrotic debris and fibrin, confirming a nonviable chronic gestation.

Postoperative recovery was uneventful, and the patient was discharged on the third postoperative day. At six-month follow-up, she remained asymptomatic, with a normal clinical examination and pelvic ultrasound showing a normal right ovary and no evidence of recurrence. She was counselled that spontaneous conception remains possible with the preserved right ovary and fallopian tube.

## Discussion

This case highlights the diagnostic challenge of CEP presenting as a large complex pelvic mass mimicking ovarian malignancy in a reproductive‑aged woman following a suspected miscarriage. The principal clinical implication is that CEP should remain within the differential diagnosis of complex adnexal masses, even when serum β‑hCG is negative.

CEP is an uncommon form of ectopic pregnancy, accounting for approximately 1%-2% of cases [5,12]. Unlike acute ectopic pregnancy, CEP follows a slowly progressive clinical course characterized by repeated minor tubal ruptures, degeneration of trophoblastic tissue, and gradual organization of hemorrhagic material into a chronic pelvic hematocele [13]. Consequently, patients often present with nonspecific symptoms such as persistent pelvic pain, irregular vaginal bleeding, or adnexal masses, frequently leading to delayed diagnosis or misclassification as other gynecologic or inflammatory conditions [4,5].

A major diagnostic pitfall is the absence of detectable serum β‑hCG. In CEP, trophoblastic tissue is frequently sparse, degenerated, or nonviable, resulting in minimal hormone production [11]. Several published reports have similarly described low or negative β‑hCG levels in histologically confirmed CEP, emphasizing that a negative pregnancy test does not reliably exclude the diagnosis [12]. In the present case, the negative β‑hCG result initially shifted diagnostic consideration toward alternative pathologies, including ovarian neoplasia.

Imaging plays an important role in the evaluation of CEP; however, radiologic findings are often nonspecific. Ultrasonography may demonstrate a heterogeneous adnexal or pelvic mass with hemorrhagic features but may not clearly distinguish CEP from tubo‑ovarian abscess, organized hematoma, endometriosis, or ovarian malignancy [9,12]. MRI offers additional advantages through superior soft tissue characterization and improved assessment of hemorrhagic components, adhesions, and lesion extent [10]. In this patient, MRI demonstrated a large hemorrhagic lesion without post‑contrast enhancement or obvious solid malignant components, findings that reduced suspicion for invasive ovarian malignancy and assisted preoperative planning. The absence of significant enhancement also argued against a tubo‑ovarian abscess, which typically demonstrates peripheral inflammatory enhancement. Nevertheless, imaging alone remained insufficient for definitive diagnosis.

The differential diagnosis in this case included tubo‑ovarian abscess, organized pelvic hematoma, and ovarian neoplasm. Tubo‑ovarian abscess typically presents with fever, leukocytosis, inflammatory symptoms, and rim‑enhancing collections on imaging. Ovarian malignancy more commonly demonstrates solid enhancing components, papillary projections, ascites, or elevated tumor markers. In contrast, CEP is often characterized intraoperatively by dense adhesions, chronic hemorrhagic material, distorted tubal anatomy, and organized hematocele formation, findings that were consistent with the operative observations in the present case [2,4,5]. These overlapping clinical and imaging features underscore the importance of correlating radiologic, operative, laboratory, and histopathological findings. Key distinguishing features are summarized in Table 2.

**Table 2:** Differential diagnosis of complex adnexal masses based on clinical, laboratory, and radiologic features β-hCG: beta-human chorionic gonadotropin, CA-125: cancer antigen 125, CRP: C-reactive protein, MRI: magnetic resonance imaging, WBC: white blood cell count

Feature	Chronic ectopic pregnancy	Tubo-ovarian abscess	Ovarian malignancy
Typical age group	Reproductive age	Reproductive age (often younger)	Predominantly postmenopausal
Principal symptoms	Pelvic pain, irregular vaginal bleeding, history of amenorrhea or recent pregnancy event	Fever, lower abdominal pain, pelvic tenderness, vaginal discharge	Often asymptomatic early; later bloating, weight loss, ascites, early satiety
Physical examination	Adnexal mass with mild or variable tenderness	Tender adnexal mass, cervical motion tenderness, fever	Fixed, irregular adnexal mass ± ascites
β-hCG	Low or negative	Negative	Negative (except germ cell tumors)
CA-125	Normal	Normal or mildly elevated	Often elevated (>35 U/mL)
Inflammatory markers (WBC/CRP)	Normal or mildly elevated	Markedly elevated	Usually normal
MRI enhancement pattern	No significant enhancement (organized hemorrhagic mass)	Peripheral rim enhancement	Solid or irregular enhancement of nodular components
MRI signal characteristics	T1/T2 hyperintense hemorrhagic mass	Complex cystic lesion with debris and fluid-fluid levels	Mixed solid-cystic lesion with enhancing components
Free intraperitoneal fluid	Absent or minimal (encapsulated hematocele)	May be present	May be present (ascites common)
Intraoperative findings	Organized hematocele, dense adhesions, tubal distortion	Purulent material, inflamed adnexa, possible rupture	Solid mass with necrosis and possible peritoneal spread
Histopathology	Chorionic villi, trophoblastic remnants, hemorrhage, fibrosis	Acute and chronic inflammatory infiltrate, abscess formation	Malignant epithelial or stromal cells
Primary treatment	Salpingectomy or salpingo-oophorectomy; methotrexate generally ineffective in chronic ectopic pregnancy	Broad-spectrum antibiotics ± image-guided drainage or surgery	Oncologic staging surgery ± platinum-based chemotherapy

Histopathological examination remains the diagnostic gold standard for CEP [5,11]. Identification of chorionic villi, trophoblastic tissue, hemorrhage, fibrin deposition, fibrosis, and tubal wall degeneration confirms the diagnosis and excludes malignancy [12]. In the present case, immature chorionic villi and intermediate trophoblast within hemorrhagic and fibrotic tissue established the diagnosis conclusively.

Surgical management remains the cornerstone of treatment for CEP. Fertility-preserving surgery may be difficult because of extensive adhesions, chronic inflammation, and distorted pelvic anatomy [2,5]. Although laparoscopy has been successfully reported in selected stable patients with smaller lesions, laparotomy may be preferable in cases involving large pelvic masses, suspected dense adhesions, ongoing hemorrhage, or uncertainty regarding malignancy [12]. The present patient required salpingo‑oophorectomy because the adnexal structures were densely adherent and normal tissue planes were completely obliterated. Methotrexate is generally considered ineffective in CEP because trophoblastic activity is minimal and the lesion largely consists of organized hemorrhagic and fibrotic tissue.

Overall, this case emphasizes the importance of including CEP within the differential diagnosis when evaluating reproductive-aged women presenting with complex hemorrhagic pelvic masses and discordant clinical, laboratory, and imaging findings.

## Conclusions

This case demonstrates CEP mimicking ovarian malignancy in a reproductive-aged woman presenting with indolent symptoms, an organized hematocele, and negative serum β-hCG levels post-miscarriage. Pelvic MRI identified the hemorrhagic lesion and excluded solid malignancy, guiding exploratory laparotomy and left salpingo-oophorectomy. Key learning points include maintaining high clinical suspicion for CEP in atypical complex pelvic masses and utilizing MRI to direct appropriate surgical management while avoiding unnecessary oncological interventions.
